# The Long-Term Survivorship and Cause of Failure of Metal-on-Metal Total Hip Arthroplasty

**DOI:** 10.3390/antibiotics14020161

**Published:** 2025-02-06

**Authors:** Hiroki Wakabayashi, Masahiro Hasegawa, Yohei Naito, Shine Tone, Akihiro Sudo

**Affiliations:** Department of Orthopaedic Surgery, Mie University Graduate School of Medicine, 2-174 Edobashi, Tsu 514-8507, Japan; masahase@clin.medic.mie-u.ac.jp (M.H.); yo-yo@clin.medic.mie-u.ac.jp (Y.N.); s-tone@clin.medic.mie-u.ac.jp (S.T.); a-sudou@clin.medic.mie-u.ac.jp (A.S.)

**Keywords:** periprosthetic hip joint infection, irrigation and debridement, antibiotic-impregnated calcium hydroxyapatite

## Abstract

Background: Complications associated with metal-on-metal (MOM) prostheses, such as adverse reactions to metal debris (ARMDs), include pseudotumor (PT) formation, metallosis, and soft tissue necrosis. High short-term failure rates have been reported for various MOM total hip arthroplasties (THAs) due to ARMDs. ARMDs in MOM THAs can potentially lead to secondary failure modes, such as dislocation or infection. Objectives: This study aims to examine the cumulative incidence of revisions due to ARMDs and periprosthetic joint infection (PJI) in primary MOM total hip arthroplasty and to compare the outcomes of ARMD and PJI cases. Methods: Between 2006 and 2011, 247 primary MOM THAs were performed on 230 patients (39 men, 191 women) with a mean age of 64.1 years. The average follow-up duration was 10.5 years. Results: Thirty-eight hips were converted to metal-on-polyethylene articulation between 1.2 and 14.7 years postoperatively (mean: 7.2 years) due to pain, swelling, infection, and/or implant failure. Eight hips (3.2%) were complicated by infection, while 30 hips (12.1%) were diagnosed with ARMDs. Rheumatoid arthritis (RA) was significantly more prevalent in patients with PJI. Preoperative C-reactive protein (CRP) levels were significantly elevated in THAs diagnosed with PJI compared to ARMD cases. Additionally, the preoperative white blood cell (WBC) counts, neutrophil counts, and neutrophil-to-WBC ratios were significantly higher in THAs with PJI, while the lymphocyte-to-WBC ratios were significantly lower. Conclusion: The incidence of postoperative infection in MOM THA cases was 3.2%, with a notable occurrence of late-onset infections. Differentiating ARMDs from PJI in MOM THA cases remains crucial.

## 1. Introduction

Total hip arthroplasty (THA) is an established treatment for managing pain and dysfunction in the hip due to progressive osteoarthritis. Over the years, numerous innovations have been developed to enhance implant stability, longevity, and clinical performance. Large-diameter heads (LDHs) and dual-mobility implants were introduced to improve stability and reduce dislocation risks. Metal-on-metal (MOM) bearings and implants with LDHs have been widely utilized for their stabilizing benefits [1]. The popularity of MOM bearing surfaces in THAs peaked in 2007, when over 30% of THA procedures in the United States employed these surfaces [2].

MOM prostheses offer several advantages, including an enhanced range of motion, reduced dislocation risks, decreased volumetric wear, and durable bearing surfaces. For younger, active individuals, concerns about wear have encouraged the exploration of alternatives like MOM, theoretically believed to exhibit lower wear rates than conventional THAs [3].

However, MOM bearings have been linked to biological complications, such as adverse reactions to metal debris (ARMDs) and pseudotumor formation [4,5]. ARMDs encompass a spectrum of disorders caused by implant wear and longevity [6]. Long-term studies on MOM THAs, specifically regarding ARMDs and revision rates, remain limited. Although MOM surfaces are now rarely used, analyzing long-term data may provide insights into their management [7].

Failed MOM THAs have been associated with higher infection rates [8]. Browne et al. [4] reported that 19% of various MOM implant revisions were due to infection. Similarly, data from the Australian Orthopaedic Association’s National Joint Replacement Registry indicated that 20% of ASR XL implant revisions were infection related [9].

Clinical testing, imaging, and aspirated cell counts may yield similar findings in aseptic reactions (ARMDs) and septic conditions, making it challenging to differentiate the two. Failures in MOM THAs may mimic hip infections, and both conditions can coexist, necessitating careful investigation [3,10,11,12]. This study investigates the cumulative incidence of ARMD- and periprosthetic joint infection (PJI)-related revisions in MOM THAs and compares these outcomes.

## 2. Results

A total of 230 patients (247 hips) were evaluated, with a mean follow-up period of 10.5 years (range, 1–16 years) in the MOM THA group. Postoperative MRIs were performed on all 232 hips. Pseudotumors (PTs) attributable to MOM articulation were observed in 80 hips (34.5%) by MRI.

In the MOM THA group, thirty-eight hips (36 patients) were converted to metal-on-polyethylene articulation between 1.2 and 14.7 years postoperatively (mean: 7.2 years) due to pain, swelling, infection, and/or implant failure. An additional hip underwent cup revision and conversion to metal-on-polyethylene articulation due to aseptic cup loosening, occurring 4 months after a fall-related trauma.

**Figure 1:**
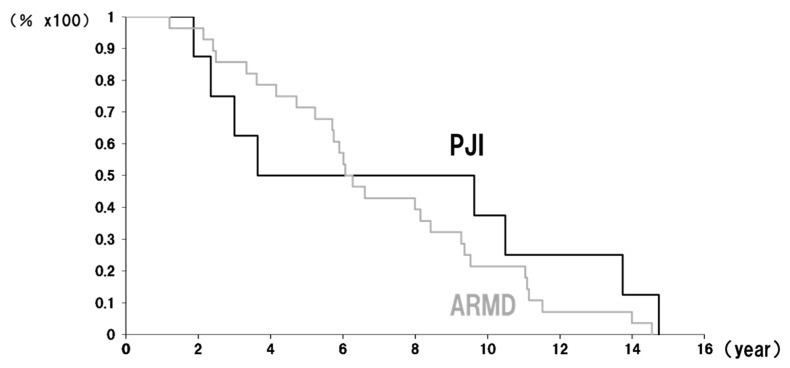
Implant survival curves at the revision endpoints for PJI and ARMDs: Black line—PJI (periprosthetic joint infection). Gray line—ARMDs (adverse reactions to metal debris).

Eight hips (3.2%) in seven patients were complicated by infection at an average of 7.4 years (median: 6.6 years; range: 1.9–14.7 years) following primary THA. These cases were diagnosed as PJIs using the Musculoskeletal Infection Society (MSIS) International Consensus Meeting (ICM) 2013 criteria [13]. PTs were identified in six of these hips. Seven hips had late-onset infections occurring more than two years after primary THA, with four cases arising after nine years. The affected hips included three in two males and five in five females. Pathogenic bacteria were identified in six hips from five patients: Staphylococcus aureus (four hips), Streptococcus anginosus (one hip), and Listeria (one hip). No causative bacteria were identified in two hips (Figure 1, Table 1).

Clinically, pain was reported in seven hips, with two presenting with cup loosening as a preoperative symptom of infection. One hip exhibited only swelling, and another demonstrated swelling and fistula formation. All patients underwent revision surgery. The revision approaches included the following: irrigation and debridement followed by modular component replacement (head, liner, S-ROM stem, and Profemur TL modular neck) in seven hips from six patients. A one-stage revision was performed for implants exhibiting osteolysis, loosening, or both. A two-stage revision was performed in one scheduled case.

PJI was treated with antibiotic-impregnated calcium hydroxyapatite in seven hips. During the follow-ups (9 months to 12 years; average: 8 years and 4 months), no recurrence of infection was observed.

Thirty hips (29 patients) were diagnosed with ARMDs, with PTs identified in 21 hips. The ARMD diagnosis was based on the presence of PTs, progressive osteolytic lesions, or both in patients with clinical symptoms. The final diagnosis of ARMDs and PJI was confirmed through intraoperative and histopathological findings.

In THAs diagnosed with ARMDs, the head, liner, modular components, and implants exhibiting osteolysis or loosening (e.g., cup and stem) were removed at an average of 7.1 years (median: 6.2 years; range: 1.2–14.5 years) after the primary THA. The revision THAs involved modular component exchange and implant replacement, converting the articulation to metal-on-polyethylene.

The timing of revision surgeries exhibited distinct patterns: ARMD-related revisions showed a progressive increase over time, whereas PJI-related revisions followed a biphasic pattern, occurring either within four years or after nine years postoperatively (Figure 1).

The patient demographics for PJI and ARMD cases are summarized in Table 2. Among the PJI cases, primary hip osteoarthritis (OA) was the indication for primary THA in 62.5% (5/8 hips), while rheumatoid arthritis (RA) accounted for 37.5% (3/8 hips). In ARMD cases, primary hip OA was the indication in 90% (27/30 hips), RA in 6.7% (2/30 hips), and the avascular necrosis of the femoral head following a post-traumatic fracture occurred in one case (3.3%).

Patient demographics were not significantly prevalent between THA patients diagnosed with PJI and those with ARMDs. The rate of PT positivity was similar between PJI cases (75%, 6/8 hips) and ARMD cases (70%, 21/30 hips). Although the preoperative CRP positivity rate was higher in PJI cases (85.7%, 6/7 patients) than in ARMD cases (44.8%, 13/29 patients), the difference was not statistically significant. However, the mean preoperative CRP level was significantly higher in PJI cases (3.89 mg/dL) compared to ARMD cases (0.41 mg/dL). Furthermore, the preoperative white blood cell (WBC) counts and neutrophil counts were significantly higher in PJI cases (Table 3).

In the non-MOM THA group, only one hip required revision surgery on the cup side due to dislocation in a case of metal-on-polyethylene THA, which occurred 11 years postoperatively. No revisions were observed in cases of ceramic-on-polyethylene THAs.

## 3. Discussion

Metal-on-metal (MOM) bearings release metallic particles ranging from nanometer to submicrometer dimensions, significantly increasing the corrosion-prone surface area when particle numbers are high [14,15]. The release of ions and particles from MOM implants is associated with severe localized biological responses, including adverse reactions to metal debris (ARMDs), metallosis, and pseudotumor formation. These complications can lead to functional impairment and extensive tissue destruction [3,16].

Pseudotumors and ARMDs are significant concerns after MOM hip arthroplasty. Some patients with pseudotumors experience severe soft tissue damage, resulting in early implant failure [17]. Pseudotumors may be asymptomatic or present with various symptoms, such as groin pain, discomfort, cup loosening, or nerve palsy [18,19]. Local inflammation mediated by wear debris and ions released from corrosion may contribute to implant loosening and bone resorption around the prosthesis [20]. Furnes O et al. reported a comprehensive analysis of the MOM implant outcomes in younger patients using uncemented fixation and found that large-head-size MOM implants were associated with an increased risk of revision after two years compared with metal-on-highly cross-linked polyethylene implants, with the effect becoming more pronounced over time [21]. In our study of the non-MOM THA group, one hip required revision surgery on the cup side due to dislocation in a case of metal-on-polyethylene THA. And no revision surgeries were observed in cases of ceramic-on-polyethylene THAs.

The deposition of these particles in periprosthetic tissue can lead to extensive necrosis, severe foreign body macrophage reactions, and pronounced cell-mediated immune responses [22,23]. The relationship between the inflammatory and necrotic changes observed in MOM periprosthetic tissues and the development of PJI remains poorly understood. While there is little evidence in the literature to support the theory that the infection risk is inherently higher in MOM THAs, some reports suggest a higher-than-expected prevalence of infection. For instance, prior studies have described a case series involving nine hips in eight patients who underwent revision surgery for local soft tissue reactions to MOM THA; three of these cases were found to have concurrent infections [3]. Similarly, Prieto HA et al. followed 124 patients who received MOM hip arthroplasty between 2006 and 2010 with a minimum follow-up of three years, identifying eight cases of acute delayed or late PJI [2].

The Australian Orthopaedic Association National Joint Replacement Registry recorded 3115 revisions of MOM THA. Among these, the most common reasons were metal-associated pathology (41.2%), implant loosening or osteolysis (27.3%), and infections (10.9%). The 10-year cumulative revision rate for infections in MOM THAs was 2.5%, compared to 0.8% for other bearing surfaces nationwide [24]. Although it remains unresolved whether MOM THAs carry a greater infection risk than other THA implants, prior studies suggest a potential elevation in risk [25]. Cobalt and chromium wear and corrosion products from MOM THAs have been reported to suppress immune responses, either hinder or enhance bacterial growth, and promote antibiotic resistance. Additionally, some metals can interfere with the efficacy of certain antibiotics against bacteria [26].

The presence of pseudotumors, along with the associated necrosis and fluid accumulation, may provide an ideal environment for acute hematogenous late PJI. A higher-than-expected incidence of PJI has been observed in cases of revision for failed aseptic MOM hip arthroplasty.

Patients with ARMDs, pseudotumors, or both typically present with generalized complaints of pain and hip dysfunction. Radiological findings often reveal cystic masses in these patients, which can be difficult to distinguish from infections [11,27]. Diagnosing local reactions in MOM total hip replacements is particularly challenging because the clinical presentation of ARMDs often mimics infection. Since treatment strategies and outcomes differ substantially, a thorough evaluation is essential for patients with painful MOM hip implants suspected of infection or ARMDs [28].

Differentiating ARMDs from infection remains challenging due to the absence of reliable and standardized diagnostic tests in most medical facilities [29]. However, this differentiation is critical for determining appropriate management strategies. Commonly used diagnostic tests, including ESR, CRP, and histopathology, are employed to distinguish septic from aseptic implant failure [30,31]. The MSIS diagnostic criteria for PJI, based on clinical, microbiological, and histological parameters [13,30], were used in this study to diagnose PJI. The final diagnoses of ARMDs and PJI were determined through intraoperative findings and histopathological confirmation.

The standard evaluation for diagnosing infected joint prostheses includes serum ESR and CRP levels. When these values are elevated or suspicion is high, a hip aspiration is performed to assess the WBC count (total nucleated cell count), white cell differential (especially the percentage of polymorphonuclear cells [PMNs]), and bacterial cultures with sensitivity testing. While nonspecific, elevated ESR and CRP levels are reliable indicators of systemic inflammation. In this study, the CRP levels, WBC counts, neutrophil counts, and neutrophil-to-WBC ratios were significantly higher in PJI cases compared to ARMD cases. In contrast, the lymphocyte-to-WBC ratios were significantly lower in PJI cases. A positive culture from hip aspiration fluid remains as the gold standard for the preoperative diagnosis of infected THAs. Even if fluid cultures are negative, elevated inflammatory markers strongly suggest revision surgery for PJI.

This study has limitations, including those inherent to any retrospective design. The sample size was relatively small, there was no control group, and the dropout rate was high. Furthermore, the data on blood cobalt and chromium levels were unavailable for analysis.

## 4. Materials and Methods

This retrospective study followed patients who underwent cementless MOM THAs at a single institution between 2006 and 2011. A total of 247 primary MOM THAs was performed using the following acetabular prostheses: 89 Pinnacle (DePuy, Warsaw, IN, USA), 108 Cormet (Corin, Cirencester, UK), and 50 Conserve Plus (Wright Medical Technology, Inc., Arlington, TN, USA). The cohort included 39 men and 191 women, with a mean age of 64.1 years and an average follow-up duration of 10.5 years (Table 4). During the observation period (2006–2011), 158 patients (159 hips) underwent cementless non-MOM THA. All primary THA patients during the observation period were included. However, twelve patients who underwent bilateral THAs during this period and received both MOM THAs and non-MOM THAs were excluded from the non-MOM THA group. After excluding these 12 patients, the patient demographics for 146 patients (147 hips) treated with non-MOM THAs are summarized in Table 5. Of these, 69 hips underwent metal-on-polyethylene procedures, while 78 hips underwent ceramic-on-polyethylene procedures, with the respective average follow-up durations of 11.7 and 10.7 years.

Ethical approval was obtained from the institutional review board, and all participants provided informed consent. The procedures adhered to the ethical standards of the institutional committee and the 1964 Helsinki Declaration and its later amendments.

### 4.1. Clinical Evaluation

The cases of revision during follow-up were reviewed retrospectively to assess the frequency and timing of revisions due to ARMDs and PJI. A comparative analysis of pre-revision test results and medical histories was performed between ARMD and PJI cases.

The diagnosis of ARMDs was based on the presence of pseudotumors (PTs), progressive OL lesions, or both in patients with clinical symptoms. The diagnosis of infection was based on clinical criteria, including the presence of a discharging sinus, purulent fluid, or pus found during preoperative hip aspiration, or positive laboratory and histopathological findings. PJI was defined as the presence of a sinus tract communicating with the prosthesis, at least two identical positive cultures, or both. PJIs were diagnosed using the Musculoskeletal Infection Society (MSIS) International Consensus Meeting (ICM) 2013 criteria. We evaluated joint-related factors, such as the presence of PTs and revision surgeries, based on the number of hip joints, while systemic factors, including patient background and blood test data, were assessed per patient.

### 4.2. Radiological Evaluation

Radiological assessments were conducted at the final follow-up or before revision surgery. Osteolysis (OL) was evaluated using the Gruen and DeLee classifications [32,33]. MRI for PT screening was performed two years postoperatively and, subsequently, every 2–3 years until the 10-year mark.

### 4.3. Statistical Analysis

The statistical analyses employed the Wilcoxon signed-rank test, analysis of variance, and Spearman’s rank correlation coefficient. A *p*-value < 0.05 was deemed to be statistically significant. The Kaplan–Meier method estimated THA survivorship, considering the time to revision, last follow-up, or death. The analyses were performed using IBM SPSS Statistics 26 (IBM Japan, Tokyo, Japan).

In conclusion, the incidence of postoperative infection in MOM THA cases was 3.2%. Differentiating MOM THA cases from ARMDs is essential to guiding appropriate management. Positive cultures from hip aspiration fluid remain the gold standard for diagnosing infected THAs preoperatively. Even when fluid culture results are negative, elevated inflammatory markers, such as CRP, WBC count, and neutrophil count, strongly suggest PJI and justify recommending revision surgery.

## Figures and Tables

**Table 1:** Revision cases for PJI.

Case	Age, Sex, and Diagnosis	BMI (kg/m^2^)	Observed PT After Primary Arthroplasty	Time to Revision	Causative Bacteria	Cup Inclination	Cup Anteversion	Symptom	Cup Revised	Stem Revised
Case1	53 F RA	16.5	6.5 years	10.5 years	Listeria	55.7	24.2	Swelling		Revised (S-ROM)
Case2	68 M RA	27.0	Not observed	3.5 years	MSSA	39.6	22.2	Pain		Revised (S-ROM)
Case2	68 M RA	27.0	Not observed	3 years	MSSA	36.7	14.1	Pain		
Case3	69 F OA	21.8	5.1 years	9.7 years	Streptococcus anginosus	57.7	3.1	Pain, Cup and stem osteolysis	2stage	2stage
Case4	66 F OA	18.9	13.6 years	13.7 years	MSSA	32.7	2.7	Swelling, Fistula, Cup and stem osteolysis	Revised	
Case5	60 F OA	22.5	2.3 years	2.3 years	no causative	42.8	11.9	Pain	Revised	
Case6	58 M OA	31.7	1.7 years	1.9 years	no causative	53.2	6.0	Pain	Revised	
Case7	63 F OA	21.4	10.1 years	14.7 years	MSSA	58.8	27.8	Pain, Cup osteolysis	Revised	

**Table 2:** Patient demographics for revisions related to PJI and ARMD.

	Revision for PJI (3.2%)	Revision for ARMD (12.1%)	*p*
Male/Female	2 patients (3 hips)/5 patients	4 patients/25 patients (26 hips)	ns
Age at revision	Mean 62.4 (58–69)	Mean 64.5 (52–69)	ns
Preoperative diagnosis	OA 5 patients RA 2 patients (3 hips)	OA 26 patients (27 hips) RA 2 patients Fx 1 patient	ns
CRP (≦0.14 mg/dL)	CRP-positive 7 patients CRP-negative 1 patient	CRP-positive 13 patients (14 hips) CRP-negative 16 patients	ns

OA—osteoarthritis; RA—rheumatoid arthritis; Fx—secondary hip OA in post-traumatic fracture.

**Table 3:** Evaluation metrics among revision surgeries for ARMDs and PJI.

Revision for	PJI (8 Hips)	ARMDs (30 Hips)	*p*
Frequency (%)	3.2%	12.1%	ns
PT-positive (%)	75%	70%	ns
**Revision for**	**PJI (7 Patients)**	**ARMDs (29 Patients)**	***p***
CRP-positive (%)	85.7%	44.8%	ns
CRP level (mean ± SD)	3.89 ± 3.06	0.41 ± 0.67	<0.005
WBC counts (mean ± SD)	8060 ± 3181	5651 ± 1357	<0.05
Neutrophil counts (mean ± SD)	5927 ± 3181	3664 ± 1280	<0.05
Lymphocyte counts (mean ± SD)	1390 ± 319	1410 ± 484	ns
Neutrophil-to-WBC ratios (mean ± SD)	71.0 ± 8.5	64.2 ± 9.4	ns
Lymphocyte-to-WBC ratios (mean ± SD)	19.2 ± 6.9	25.5 ± 8.0	ns

ARMDs—adverse reactions to metal debris; PJI—periprosthetic joint infection; SD—standard deviation; ns—not significant.

**Table 4:** Demographics of patients undergoing MOM THA.

Male/Female	39 Patients/191 Patients		
Age (years)	Mean 64.1 (34–85)		
Preoperative diagnosis	Osteoarthritis (OA)	223 hips	
	Rheumatoid arthritis (RA)	15 hips	
	Osteonecrosis (ON)	7 hips	
	Secondary OA in post hip fracture	2 hips	
Implant	Pinnacle	82 patients	89 hips
	Cormet	98 patients	108 hips
	Conserve plus	50 patients	50 hips
Follow-up periods	Mean 10.5 years		

**Table 5:** Demographics of patients undergoing non-MOM THA.

	Metal on Polyethylene	Ceramic on Polyethylene
	68 patients/69 hips	78 patients/78 hips
Age (years)	Mean 60.8	Mean 64.9
Preoperative diagnosis		
Osteoarthritis (OA)	57 hips	72 hips
Rheumatoid arthritis (RA)	5 hips	2 hips
Osteonecrosis (ON)	3 hips (2 patients)	
Rapidly destructive coxarthrosis	2 hips	3 hips
Secondary OA in infected hip	2 hips	
Pigmented villonodular synovitis		1 hip
Follow-up periods	Mean 11.7 years	Mean 10.7 years

## Data Availability

Data are contained within the article.
